# Supplementary material for: Multivariate prediction of temper outbursts in a sample of youth enriched for irritability using ecological momentary assessment data: A registered report
Source: PLoS One. 2025 Mar 18;20(3):e0289235. doi: 10.1371/journal.pone.0289235 (PMC11918405; doi:10.1371/journal.pone.0289235)
Supplement: S1 Appendix — (DOCX) [file pone.0289235.s001.docx]

**S1 appendix**

Results of power analysis


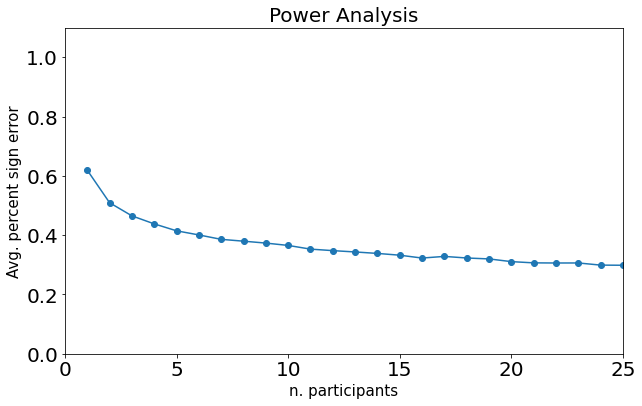


The figure above shows the power as a function of the average number of ratings collected.  The blue lines shows the power in terms of the average proportion of coefficients with incorrectly estimated signs using regularized balanced logistic regression. To compare the accuracy of using a 10-participant confirmation set versus a larger 15-, 20-, 25-, or 30-participant sample size, we display the simulation-derived power in the below table.

| Sample size (participants) | Avg. number of ratings | Avg. percent sign error | Simulation standard error of avg. percent sign error (from 1000 simulations per sample size) |
| --- | --- | --- | --- |
| 10 | 149.396 | 0.366 | 0.003 |
| 15 | 225.147 | 0.323 | 0.003 |
| 20 | 300.794 | 0.311 | 0.002 |
| 25 | 374.531 | 0.299 | 0.002 |
| 30 | 465.116 | 0.287 | 0.002 |

With n=10 participants, we have an average 37% of variables in the regression (this includes both lag-1 and lag-2 terms) with incorrectly estimated signs compared to the SGT. This error rate drops by about 4% when we add another 5 participants (n=15). If we repeatedly add 5 participants, each additional 5 participants only drops the average error rate between 1-2% up to n=30. We did not have enough participants in the training set to simulate n=35 participants.

Discussion for power analysis

What this suggests in terms of the optimal sample size depends on our subjective assessment of the cost vs. reward tradeoff of collecting more participants.  We are mindful of the difficulty for young participants struggling with emotional disorders to answer numerous, potentially emotionally evocative questions repeatedly as required by our protocol, so in planning the study we want to minimize participant burden.

However, another consideration to selecting the sample size is the potential scientific contribution beyond the specific population of children with irritability and their families, as well as the ethical duty of scientists to ensure the reproducibility and trustworthiness of scientific work.

There was a consensus that when we take into account both the clinical and methodological considerations, the more conservative would be 25 participants even though a case could be made to justify 15 participants ratings.

Our consensus decision is to take the average of these two extremes, and therefore our new plan is to stop collecting participants for this analysis once we have collected 20 participants (who complete the study without dropping out), which results in around an 300 ratings on average.  For this number of ratings, assuming that  the same proportion of ratings will contain outbursts as in the exploratory sample (10%), we expect to see 30 temper outbursts on average.
