# Supplementary material for: Multivariate prediction of temper outbursts in a sample of youth enriched for irritability using ecological momentary assessment data: A registered report
Source: PLoS One. 2025 Mar 18;20(3):e0289235. doi: 10.1371/journal.pone.0289235 (PMC11918405; doi:10.1371/journal.pone.0289235)
Supplement: S2 Appendix — (DOCX) [file pone.0289235.s002.docx]

**S2 Appendix**

Time lag selection for regularized logistic regression

We fit the regularized logistic regression model with lags 1, 2, and 3 and obtained the following results.

| Lag | AUC on test set | std. dev. of AUC under bootstrap (B=10) |
| --- | --- | --- |
| 1 | 0.73 | 0.052 |
| 2 | 0.78 | 0.063 |
| 3 | 0.77 | 0.065 |

Given these results, it would be reasonable to either adopt the lag-2 model, which obtained the highest AUC for test data, or continue with the lag-1 model, since the AUC of the lag-2 model is within less than one standard deviation of the AUC of the more parsimonious lag-1 model.  We would not consider the lag-3 model since it has lower AUC than the less complex lag-2 model, which is indicative of overfitting.

To decide between the lag-1 and lag-2 model, we examined the coefficients of the lag-2 model (shown in table 3 of the paper, also replicated above).  We were struck by the matching signs and similar magnitude between the lag-1 and lag-2 coefficients for each time-varying variable.  Furthermore, the pattern of whether the magnitude stayed about the same going from lag-1 to lag-2 or shrunk seemed to align with prior knowledge about which variables were phasic versus tonic.  This seemed scientifically interesting enough to switch our focus to the more complex lag-2 model.

Time-of-day-specific modeling analysis

We compared the time-specific model AUC to the time-non-specific model AUC.  We used the lag-1 logistic regression model for this analysis, since the data size would be much smaller when restricting the training set to individual times of day.  Since the AUC of the two models may differ by chance, we computed the standard deviation of the time-specific model AUC using bootstrap resampling (B=1000) of the training data.  Please note that the standard deviation reported is an underestimate of the true variation that would be observed in a replicate data set, as it only reflects randomness in the training set and not the test set.  The results are as follows.

Table A

| Time of day being predicted | Number of outbursts | Time-specific model AUC (std. dev) | Time non-specific model AUC |
| --- | --- | --- | --- |
| Morning | 2 | 0.94 (0.10) | 0.94 |
| Afternoon | 4 | 0.70 (0.06) | 0.66 |
| Night | 7 | 0.70 (0.13) | 0.72 |

Although we see some differences in AUC between morning, afternoon, and night, the low number of outbursts per time of day (7 at most) prevents us from drawing reliable conclusions about the comparative predictability of the different times of day.  Note also that the variation in the time period between ratings and the unequal frequencies of rating by time of day pose additional obstacles to comparing the three times of day.

Overall, while some of these findings are interesting, we decided that the extremely low effective sample sizes (as determined by number of outbursts) more or less confirmed our initial hunch that analysis of specific times of day would be impractical for this data set.  We will include these analyses in the supplement, but note that these were done in response to the reviewer's query and that we do not consider the sample sizes to be adequate for drawing any conclusions from these analyses.

Model coefficients

Lag-1

| Variable (lag) | Coefficient (no lag) | Coefficient (lag-1) |
| --- | --- | --- |
| happy |  | -0.20 |
| unhappy |  | 0.14 |
| grouchy |  | 0.14 |
| frustrated |  | 0.13 |
| angry |  | 0.27 |
| mood_changed |  | 0.09 |
| worried_now |  | 0.13 |
| been_worried |  | 0.14 |
| tired |  | 0.10 |
| age | -0.08 |  |
| female | 0.22 |  |
| Night | 0.02 |  |
| Monday | -0.11 |  |
| Tuesday | -0.07 |  |
| Wednesday | -0.02 |  |
| Saturday | -0.04 |  |
| Sunday | 0.01 |  |
| *(intercept)* | -0.16 |  |

Lag-2

| Variable | Coefficient (no lag) | Coefficient (lag-1) | Coefficient (lag-2) |
| --- | --- | --- | --- |
| happy |  | -0.20 | -0.08 |
| unhappy |  | 0.10 | 0.12 |
| grouchy |  | 0.13 | 0.07 |
| frustrated |  | 0.08 | 0.03 |
| angry |  | 0.25 | 0.22 |
| mood_changed |  | 0.04 | -0.01 |
| worried_now |  | 0.11 | 0.11 |
| been_worried |  | 0.08 | 0.09 |
| tired |  | 0.05 | 0.07 |
| age | -0.08 |  |  |
| female | 0.17 |  |  |
| Night | -0.01 |  |  |
| Monday | -0.11 |  |  |
| Tuesday | -0.10 |  |  |
| Wednesday | -0.01 |  |  |
| Saturday | -0.06 |  |  |
| Sunday | 0.02 |  |  |
| *(intercept)* | -0.16 |  |  |

Lag-3

| Variable | Coefficient (no lag) | Coefficient (lag-1) | Coefficient (lag-2) | Coefficient (lag-3) |
| --- | --- | --- | --- | --- |
| happy |  | -0.18 | -0.06 | -0.06 |
| unhappy |  | 0.07 | 0.09 | 0.09 |
| grouchy |  | 0.09 | 0.04 | 0.04 |
| frustrated |  | 0.04 | -0.00 | -0.00 |
| angry |  | 0.23 | 0.16 | 0.16 |
| mood_changed |  | 0.02 | -0.02 | -0.02 |
| worried_now |  | 0.09 | 0.10 | 0.10 |
| been_worried |  | 0.06 | 0.07 | 0.13 |
| tired |  | 0.05 | 0.05 | 0.04 |
| age | -0.09 |  |  |  |
| female | 0.12 |  |  |  |
| Night | -0.00 |  |  |  |
| Monday | -0.11 |  |  |  |
| Tuesday | -0.07 |  |  |  |
| Wednesday | -0.04 |  |  |  |
| Saturday | -0.07 |  |  |  |
| Sunday | 0.02 |  |  |  |
| *(intercept)* | -0.16 |  |  |  |
