# Supplementary material for: Multivariate prediction of temper outbursts in a sample of youth enriched for irritability using ecological momentary assessment data: A registered report
Source: PLoS One. 2025 Mar 18;20(3):e0289235. doi: 10.1371/journal.pone.0289235 (PMC11918405; doi:10.1371/journal.pone.0289235)
Supplement: Attachment — Submitted filename: Response to reviewers.docx [file pone.0289235.s003.docx]

**DEPARTMENT OF HEALTH & HUMAN SERVICES** Public Health Service

National Institutes of Health

National Institute of Mental Health

Functional Magnetic Resonance Imaging Facility

Bethesda, Maryland 20892

Feb 29, 2024

Rebuttal letter

To the academic editor and reviewers:

We are thankful to the academic editor and the reviewer for their insightful comments.  In addressing these comments, we were invited to think more deeply about the motivation of this work and the particular choices we made, and to perform additional analyses to justify our decisions.  In some cases, we even discovered new findings that spurred substantial changes to our paper.  We think these changes greatly improve the paper, and so we are profoundly grateful to the editor and reviewer for stimulating these developments.  We also hope that our responses and our additions to the paper will address the important concerns raised but also improve the readability and utility of the paper for readers.

Response to academic editor

*"The AUC of the random forest is mentioned to be .54 and is the reason given for not using it for further analysis. No AUC is given for the logistic regression for comparison. "*

Thank you for pointing this out. We found out that we could include an additional 8 training participants (not new data--these were 8 out of the 54 previously described participants) and this increased the AUC of random forest to 0.70. The AUC of the lag-1 logistic regression increased to 0.73 with the additional participants and we report this in the S2 appendix. Please note however that we have switched to a lag-2 logistic regression model in this revision, which has an AUC of 0.78.

*."...Table 3. While the weights for each variable is given, its also normal to provide p-values corresponding to each variable, to demonstrate which variables are significant at a particular alpha level."*

Thank you for bringing this to our attention, as we generally agree that reporting of p-values or other forms of uncertainty quantification are essential for the reporting of statistical modeling results. For the Bayesian model selection we used, there is no existing method to provide frequentist p-values.  However, it is possible to provide Bayesian posterior credible intervals for the parameters, which have similar properties to frequentist confidence intervals (Albers et al. 2018).  In section 3.4.2, we describe this approach.  We included these credible intervals in the updated Table 3.

Response to reviewer 1

*"...from the current Introduction it is unclear to me what is already known on this topic from clinical practice and theory in this area. Are there any existing (treatment) models that have been described that this research directly feeds into or expands? Without such context it is hard for me to judge to what extent this research design is a worthwhile way to investigate this topic."*

We agree with the importance of indicating existing research. We have expanded the list of cited references which we discuss in the introduction.

*"...he proposed analyses only adopt a lag-1 design (section 5.1.2.). Lag-2 and lag-3 might still contain highly relevant predictive information. Have the authors considered examining other lags?"*

Thank you for this excellent suggestion!  In response to it, we fit the regularized logistic regression model with lags 1, 2, and 3 as described in section 3.4.2.  Based on these results, we have revised the manuscript to pre-register the lag-2 logistic regression model instead of the lag-1 logistic regression model.

*Relatedly, the prediction from the last beep of the day (e.g., day 1 beep 3) to the first beep of the next day (day 2 beep 1; overnight) might be qualitatively different from the association between beeps on the same day. If I understand correctly, these overnight lags are left unaltered. Have the authors considered how the inclusion of overnight lags might affect their model results? I consider it pertinent for the authors to make their considerations explicit in the text, as well as mention this in the limitations.*

*Moreover, unequal spacing between the other within-day beeps may also affect the predictive ability of closer-spaced beeps (e.g., afternoon to evening is likely closer than morning to afternoon). Please add some discussion on this.*

Thank you for these excellent questions on the number of lags to include in the model, as well as the effects of different times of day.  We conducted additional experiments on these questions, and include results and discussion in the S2 appendix. As a result of these investigations, we decided to switch from lag-1 to lag-2 regularized logistic regression, which we describe in section 3.4.2.

We have included the following text in section 6.

"One limitation to our study is that all time periods (morning, afternoon, night) are treated equivalently by our modeling approach.  However, in practice the delay between night and morning is the longest, and while the delay between afternoon and night is usually longer than the delay between morning and afternoon, there is some variability as participants are free to choose when to complete the prompt within the allowed window.  Therefore, it is plausible that there could be differences between the three times of day in terms of the optimal prediction rule.  However, when we attempted to fit time-of-day-specific prediction models, we saw no statistically significant difference in the performance of time-of-day specific models and the model that we developed based on all times of day (see S2 Appendix).  Yet, given more data it might be possible to develop time-of-day-specific classifiers for differing times of day that would outperform a time-of-day-general classifier."

*Another point that I think requires further discussion is the fact that the proposed study aims to find group-level predictors, for within-person processes. In the Introduction, the authors already speculate about the potential for clinical applications and early warning systems through apps. However, this all rests on the assumption that their model can find generic factors from across a small group of individuals that will apply well enough to individual cases. I would like to see a deeper embedding of this in the introduction and discussion. Could the authors describe why they think it is likely that they would find generalizable results with their method?*

You are right, it is important to explain why we decided to predict only using population data and not customizing for individuals.  In summary, only a population-level approach allows immediate generalizability to a new user without requiring a period of initial data collection. We discuss this in greater detail within a discussion of individualized versus population-level prediction approaches in section 3.3.

*Some comments on section ‘2.3.2. Sample size’. I would like to see a more detailed explanation of the target sample size. Based on the description it seems that approximately 40 outbursts occurring in the span of 400 measurements is considered sufficient to run the confirmatory analyses. Can the authors provide further explanation and references to support that target number? I am satisfied that the 10% expectation comes from their own preliminary data but would like to see more justification for the chosen number of observations.*

Thank you for raising the important issue of sample size justification.  Originally, our estimate of 400 completed EMA ratings was based on intuition gained from working with the preliminary data.  However, in response to the reviewer we conducted a more rigorous power analysis to check if our intuition was well-calibrated.  We describe this power analysis in section 2.3.3.

*Also, there is no mention of the within-person and temporal dependency that these observations have, which will limit the predictive contribution of a given observation. Since some children will have no outbursts, and some may have many, this influences how much information they can draw from each person. And, since the model aims to predict next-moment temper outbursts, the beep prior to an event is almost as important as the beep with the outburst itself. Please make note of this in the manuscript and how it has affected the design choices.*

You have raised an insightful question on the choice of whether to use variables concurrent with the predicted temper outburst.  We have clarified our analysis choices and how they are tailored to the goal of developing an intervention system in section 5.1.2, where we comment that "[t]he variables coincident with a temper outburst are not used in the prediction, because given the design of the EMA application, temper outbursts will typically only be recorded after they have already occurred.  Hence, such measurements would not be available to an early warning intervention system."

*In section 4.1. the authors refer readers to their previous manuscript for the rationale behind the variables. I appreciate that paraphrasing methods sections from previous work can be tedious – especially when direct replication is the goal – and I understand that a lot of overlap exists between their earlier work and the current protocol. However, a direct explanation of the rationale for the chosen variables seems important enough. Please briefly summarize the chosen conceptualization for your measures.*

You are right, it is important for us to explain the conceptualization of our measures.  Our prompt design is based on the differentiation of irritability into tonic and phasic irritability, which we explain in the introduction.

*- The authors describe the sample as “a group of clinically-referred youth enriched for symptoms of irritability and temper outbursts”. I do not understand what the word ‘enriched’ is supposed to mean in this context, perhaps it is a jargon term. Please consider clarifying this language.*

Thanks for noting our use of jargon, and consequently a way to improve the readability of our paper for a more general audience.  We have rewritten that sentence of the abstract to read, "Here, we provide the rationale for a study to test the performance of a classifier trained to predict temper outbursts in a group of clinically-referred youth presenting with symptoms of irritability and temper outbursts."

*- The authors describe the total number of EMA time points in their preliminary sample, and intended data collection. Since the expected number of temper outbursts is crucial to the study, the authors could consider making note of that too.*

Thank you for bringing this to our attention.  We now note the number of expected outbursts in our intended data collection.

*§ 2.1*

*- The authors include a parenthetical in their hypotheses to say “Thursday and Friday being considered as one category.” I have found no clear explanation for this anywhere in the manuscript. If this is based on their prior work, they could simply add that here. If it is due to methodological limitations, please clarify that.*

Indeed, we did not include any explanation for this methodological decision, and it is important for us to explain how this was not an arbitrary choice but in fact was a consequence of the feature selection procedure we used (described in section 3.3).  In fact, the reason why Thursday and Friday end up being considered as one category is because our feature selection procedure eliminated the dummy variables for Thursday and Friday.  We now include this explanation in section 2.1.

*§ 2.3.1. Explanation of existing data*

*- Could the authors make explicit what they considered ‘not enough data’ for inclusion?*

Thank you for this excellent question!  We included the following text in section 2.3.1.  "Three were excluded for having fewer than 3 completed EMA ratings during the recording period.  (For non-machine learning studies, the inclusion criteria for rate of complete ratings would typically be more strict (e.g 7/21), but we used a less strict inclusion criteria out of the desire to examine if the prediction could work even on people with low compliance.  We considered 3 ratings out of 21 to be a minimal criterion to evidence engagement.)"

*- In Table 1, the mean number of complete ratings are reported. Please also report the range and SD.*

Thank you for this valuable suggestion!  In the process of computing the standard deviations, we noticed that we reported incorrect numbers for the number of prompts used.  We now report the corrected average number of complete prompts and the standard deviation in Table 1.  Incidentally, we now consistently use the word "prompt" to refer to the data collected from a single participant from one out of three daily sessions ather than "rating".

*§ 2.3.2.*

*- The aims for the data collection are listed here, with the age and gender distribution listed at 3 decimal precision. I found this rather confusing initially, especially because the values differ from those reported in the quoted paper and Table 1 reports only age at one decimal precision. Please make explicit here that these numbers are based on the values found in the preliminary data, and consider rounding them up.*

Thank you for bringing this formatting issue to our attention.  We have decided to use 3 decimal precision (whenever available) throughout the paper for the sake of consistency.  Also, we have included the following text in section 2.3.2.

"Note that the values in Table 1 are based on preliminary data, and hence differ from the statistics in Naim et al. 2021."

*- “The means of planned recruitment and exclusion criteria will be similar to those…” Are they similar or identical. If similar, please be explicit in what will be changed, have any steps been updated since the prior data collection?*

Good question. They are identical, and this is now indicated in section 2.3.2.

*- Note that the next section on Sample size is also labeled 2.3.2., please correct.*

Thank you for bringing this to our attention.  We have corrected the section numbering.

*§ 3.5*

*- I found the color scale of Figure 1 to be a little hard to read, given that it lacks a clear differentiation around 0. The figure note describes a ‘strong negative correlation’ for some of the variables, but I found the dark purple of 0 and near-black of -0.2 to not be very differentiable, and found this negative end of the scale additionally tricky to judge given much wider range of positive correlations. Consider replotting this with a diverging color scale (e.g., see here: https://r-graph-gallery.com/38-rcolorbrewers-palettes.html)*

Thank you for this suggestion!  We have changed the color scale of Figure 1 to a diverging color scale, and indeed it seems much easier to interpret compared to the previous version of the figure.

*§ 4.1 Measured variables*

*- Please explain the chosen use of capital letters in Table 3 and the description of the variables in section 4.1. Were the prompts presented literally with those words capitalized, or were they perhaps presented in bold? Some further description is advised.*

Thank you for bringing this lack of explanation to our attention.  Yes, the prompts were presented literally with those words capitalized and we have noted this in the table caption.

We will greatly appreciate feedback on whether we have addressed most of the concerns raised by the academic editor and the reviewer, and we look forward to any additional questions, suggestions or comments.

Sincerely,

Charles Zheng, Ph.D.

Machine Learning Team

Functional Magnetic Resonance Imaging Facility

National Institute of Mental Health, National Institutes of Health

9000 Rockville Pike, Building 10, Room 3D39

Bethesda, MD 20892-2670 USA

E-mail: [charles.zheng@nih.gov](mailto:charles.zheng@nih.gov)
