# Supplementary material for: Multivariate prediction of temper outbursts in a sample of youth enriched for irritability using ecological momentary assessment data: A registered report
Source: PLoS One. 2025 Mar 18;20(3):e0289235. doi: 10.1371/journal.pone.0289235 (PMC11918405; doi:10.1371/journal.pone.0289235)
Supplement: Attachment — Submitted filename: Response_to_reviewers_auresp_3.docx [file pone.0289235.s005.docx]

**DEPARTMENT OF HEALTH & HUMAN SERVICES** Public Health Service

National Institutes of Health

National Institute of Mental Health

Functional Magnetic Resonance Imaging Facility

Bethesda, Maryland 20892

Oct 28, 2024

Response letter

To the academic editor and reviewers:

We are thankful to the academic editor and the reviewer for their insightful comments.  In response, we have re-organized the structure of the paper, greatly improving its readability.

Response to academic editor

*The reviewers, particularly reviewer 3, have given extensive comments relating to the structuring of the manuscript. Despite changes made during the previous round of revision reviewer 3 still feels the manuscript could benefit from substantial restructuring and clarifications. In addition, please also address the point raised by reviewer 2 on how the present manuscript relates to reference [15].*

Indeed, these comments on changing the structure of the paper were very helpful, and we have re-organized the paper to be organized into separate sections for the preliminary data already collected and the confirmatory data that is planned to be collected. We also clarify the relation of the manuscript to Naim et al. 2021, as we elaborate below in our response to the reviewer #2.

Response to reviewer #2

*Please include the study method (observational, protocol) in the title*

Thank you for the suggestion. We have edited the title to "Multivariate prediction of temper outbursts in an observational study of youth enriched for irritability using Ecological Momentary Assessment data."

*I am confused about how this is a protocol when the study has one already, cited as [15], or whether this is a preliminary investigation.*

Please note that we incorrectly cited [15] for the data collection protocol. We have revised the paper to reflect the correct citation, [11]. We have added a new section on pages 4-5, "1.2. Related Work" in which we clarify the relation of this study to [11]. This is an important point that we neglected to make fully clear in the previous version.

"Naim et al. 2021 [11] describes an experimental design for testing the efficacy of exposure-based cognitive behavior therapy for severe irritability in youth. As part of the experimental design, [11] ecological momentary assessment data was collected, both from parents/caregiver and children. We use the same protocols for recruiting participant families and obtaining child EMA data as [11]. However, our protocol is observational in nature -- we do not collect data from treated patients. Furthermore, our goal is to assess the feasibility of forecasting temper outbursts from EMA data, rather than assessing the effectiveness of any treatment."

*The tense jumps between past and present and the method section describes both a past study and a future study. If this is a protocol study, then it might make more sense to present the new protocol for the ongoing/confirmatory study (assuming that it is different from 15) and the planned analyses, and then include the preliminary analyses as a result. I am also unclear as to whether the new findings (i.e., Our initial evaluation provided encouraging evidence for the possibility of predicting the presence of a temper outburst based on individual’s momentary clinical responses…) is suitable for a protocol.*

We are extremely grateful for the suggestion by reviewers #2 and #3 to re-organize our paper by clearly separating the preliminary data analysis and the protocol for confirmatory data (please see pages 6-20 for preliminary data analysis and pages 20-29 for protocol). We have re-structured our paper and think that it should read much more clearly now.

*• Please avoid long paragraphs of quotes as per the method.*

We are not sure why the reviewer is opposed to long quotes. There is no copyright issue, since the quoted text is from a published paper which we cite and believe it help the reader by giving a more context and relevant information.

*• The rationale for why day of week and time of day should relate to anger belongs in the introduction*

Thank you for this suggestion. As we elaborate further in our response to reviewer #3, question 5, we have added our rationale for including these measures in the introduction.

*• I am unclear as to why no other exploratory analyses will be conducted with the new dataset?*

Thank you for this question. Our initial purpose of the confirmatory dataset is primarily to validate the predictive performance of the classifier developed using the preliminary dataset. However, there is a hypothetical scenario where the performance on the new dataset was drastically different than the performance on the preliminary dataset. Hence, we have now included these potential post-hoc diagnostic analyses and post-hoc analyses of inter-individual variability in the new section 3.3.4., Exploratory Analyses of the revised manuscript, on pages 27-29.

Response to reviewer #2

*This is an interesting approach with potential translation into clinical practice at long term that employs EMA data to prospectively predict the occurrence of temper outbursts in children and adolescents with (supposedly) elevated levels of irritability. I have revised a revised version of the original submission. My comments are based on the revised version and the responses given to the editor and the referee. Overall, I found that this work needs to be much clearer, transparent, and streamlined. Currently, it is hard to read due to the way it is structured, since the first sections refer to relevant information that is not presented until later in the manuscript. That the text pivots back and forth between the preliminary data and the confirmatory data make is extremely confusing. As asked by the previous referee, but not addressed, I think it would be good referring less to the previous paper, and provide more information in the current one to make it more understandable and a stand alone piece. Please see my comments below:

1. First, the manuscript needs restructuring to be understandable. My suggestion is moving the whole point 3 (Classified development using preliminary data) before point 2. In addition, the current point, 2.3.1 (Explanation of existing data) should be merged under current point 3 (which, as I said, I think it should be point 2). Otherwise, talking about confirmatory data before describing the preliminary data makes it so hard to read. Moreover, you later talk about the confirmatory data again, repeating information in some instances. It could be simplified all together. I understand authors are following the OSF Pregistrered reports template, but presenting all related to the preliminary data before presenting anything else would make a huge difference. Mostly because all the hypotheses, selection of predictors, analyses etc… are a result of your preliminary data.*

We are extremely grateful for the suggestion by reviewers #2 and #3 to re-organize our paper by clearly separating the preliminary data analysis and the protocol for confirmatory data. We have re-structured our paper and think that it should read much more clearly now.

*2. In the abstract, it is said that the preliminary data consisted on 57 participants with 1197 time points, but this is not accurate. The authors describe how 3 participants were excluded because they had less than 3 prompts (out of 21) completed. Later in the methods the authors mention that “Data consisting of 1134 prompts from non-excluded participants was included”, this suggests that they had 0% of missing. Is that correct? It is hard to believe that 3 participants had less than 3 prompts and the other participants had all 21 completed. Please report % of missing prompts.*

Thank you for this suggestion! You are correct, in the abstract we include the excluded participants. We have now revised it to reflect only the included participants. As for the remaining 1134 prompts, there were 202 which were completely missing, leaving 932 prompts. We now report the number of non-missing prompts for included participants in the abstract and the percentage of missing prompts in table 1a on pages 9-10.

*3. In the current manuscript, it is not until second Table 3 that the reader finds out how the key variables used in this study were collected. My suggestion is, when moving section 3 to be section 2, under data collection, describe data collection procedure using EMA (as you have it under 2.3 Design plan> study design) and then describe thoroughly how each of the variables was measured, as in the second Table 3, and as in 4.1 Measured variables. This explanation of the variables should go way earlier before you explain any results or models, or analyses. Also, the authors should be explicit in that there are some variables that are momentary, but many others are collected retrospectively asking “SINCE the last beep…. This is important. In the models, which variables are used to predict a Temper oubtursts reported since the last prompt? The, for example, change in mood since the last prompt, or the change in mood since the last prompt from the previous prompt? Be clear about this.*

These are great suggestions! We agree with the idea of describing the details of the collected variables in proximity of the description of the data collection protocol. Hence, we now have moved the previous section 4.1 to section 2.2.1, on pages 10-15. Since the previous table 3 is redundant with the description in 2.2.3, we have deleted the table. We have also now clarified the timing of the variables used to predict a temper outburst in section 2.3. Please see our response to your question 6 for this point.

*4. There are two Tables 3. I would remove the second table. If variables are well described well in advance as asked, that table is not necessary.*

Agreed -- we have removed the second table.

*5. The referee says “In section 4.1. the authors refer readers to their previous manuscript for the rationale behind the variables. I appreciate that paraphrasing methods sections from previous work can be tedious – especially when direct replication is the goal – and I understand that a lot of overlap exists between their earlier work and the current protocol. However, a direct explanation of the rationale for the chosen variables seems important enough.*

Thank you for this suggestion. We appreciate that adding the rationale in the current paper is appropriate and improves the readability. We have added those details in section 2.2.1 on pages 10-15, which was previously section 4.1.

*Please briefly summarize the chosen conceptualization for your measures.” However, this comment is not addressed. Yes, the authors make the differentiation between phasic and tonic irritability in the introduction, but still there is no rationale for WHY these variables and not others were selected in the first place to be tested for potential predictors of temper outbursts. What is the evidence for each of these variables? What led the authors to include time of day, or day of the week as a potential predictor? And sex? And age? And happiness? And anxiety? And so on… This should be included either in the introduction or at least, in the methods when describing the variables earlier in the manuscript.*

These are excellent questions, and we have revised the introduction to discuss the background research underpinning our selection of these measures. Specifically, on pages 3-4, we now state:

"Previous work has demonstrated the feasibility of predicting symptomatic behavior from psychological traits [12, 13]. Prior research has demonstrated significant differences in the presentation and manifestation of anger and aggression by sex assigned at birth [33, 34]. Extensive prior work has shown significant changes in the trajectory of irritability by age [36, 37]. For example, while temper outbursts are normative during the preschool years, as prefrontal cortex and emotion regulation improves during school age years (i.e., 6-10), normatively temper outbursts decrease [37]. Then, during early adolescence, irritability often increases again during puberty [35]. This increase in anger and irritability during puberty also further interacts with sex assigned at birth [34].

Clinical interactions and prior implementation research have also indicated a pattern of anger and irritability that follows specific temporal relations, including time of day (e.g., post-doc decreased sleep [31], and over the course of the school week [30]. From a clinical perspective, we are aware that negative valence, high arousal emotions (e.g., anxiety and irritability) often co-occur [38] and are distinct from high arousal positively valence emotions (e.g., happiness, excitement). However, it remains unclear the temporal relationship between high arousal positive and negative emotional states [9], as they present, dynamically in vivo and specifically how these other emotions specifically relate to the later presentation of acute temper outbursts."

Furthermore, we have included rationale for each of our individual measures on pages 10-15 in section 2.2.1., "Measured variables".

*6. Related to point 3 and 5, it is said that “The variables coincident with a temper outburst are not used in the prediction, because given the design of the EMA application, temper outbursts will typically only be recorded after they have already occurred. Hence, such measurements would not be available to an early warning intervention system.” Depending how predictors are included in the model, this statement would also apply to all variables collected with “SINCE the last beep..” including irritable mood, frustration, anxious affect, anxious avoidance, grouchy and change in mood.*

We appreciate the reviewer's careful attention to the timing of the variables collected, and we see that the paper could benefit from increased clarity on the timing of the data collection and when prediction could potentially occur within an intervention system. We have therefore added a paragraph in section 2.3. that addresses the timing of the variables used for the outburst prediction. Specifically, on pages 15-16, we state:

"Since one of our goals is to test the feasibility of developing an early warning and intervention system based on EMA data, we will structure the classifier to act on data that would be available to predict an outburst before it occurs. The EMA data is collected three times a day, and during each collection period, all the questions are collected in the same short session. This includes both questions that ask for the state of the respondent (e.g. anxious, frustrated, etc.) since the time of the last beep, as well as questions that ask for the respondent's momentary state (e.g. momentary anger, hunger, etc.). Both may be predictive of an impending outburst. "

*7. The authors keep referring to paper [15] but I think they should be referring to paper [11] instead. Am I right? Or [15] is correct?*

You are correct, thank you and reviewer #2 both for bringing this to our attention. We mistakenly cited [15] rather than [11] since they were by the same first author and same publication year. We have now corrected these citations.

*8. Table 1 is odd. I would expect this table to be the first part of the results of current point 3.4. This table should be intended to provide descriptive statistics of the participants, indicating Mean and SD for continuous variables, and number of observations and percentages n(%) for categorical variables. There is no heading. Include race/ethnicity information, and other sociodemographic variables if available. We need to know what type of participants the manuscript is analyzing. Include diagnoses too.*

Thank you for these suggestions! We have collected ethnicity and diagnostic information for our participants, and these are now displayed in Table 1a on pages 9-10 (previously Table 1), which is now organized by headings such as "Ethnicity", "Diagnosis", etc.

*Ideally, the table should include descriptive statistics for all variables employed in the models.*

We agree with the reviewer’s suggestion that this added information is valuable. Hence, we have added a new table, Table 1c on pages 11-12, that contains these statistics.

*The most important are temper outbursts, but the information provided for these is unclear (Mean and SD of frequency?). What is the denominator of such frequency? I would be transparent; report Mean and SD of temper outbursts, and then mean and SD of frequency (I believe is the number of temper outbursts/number of prompts completed). Also report the range across participants.*

Thank you for these detailed suggestions! We agree that presentation of our temper outburst statistics should be clarified. We previously presented the mean and SD of temper outburst frequency across participants (overall, male, and female). The denominator of the frequency would be the number of completed ratings, which varies by participant. Now, following your suggestion, we also report the range (the min and max) frequency by participant. These can be found on table 1b in pages 10-11.

We also considered reporting the average outburst statistics, but decided not to, since the means of outbursts would be very similar to the means of frequencies by participant.

*9. The previous information is important to interpret the results of the current paper and for the following; It is interesting to see the seemingly low number of temper outbursts. For example, the authors could not estimate robustly the effects of time of day because low number of outbursts per time of day. I wonder whether there were enough number of temper outbursts per day of the week to properly test the effect of these. This is the kind of information that should be reported on Table 1.*

We appreciate your attention to the time-of-day effects on frequency of outbursts upon which we briefly commented. We have included the requested statistics in the new Table 1b in pages 10-11.

*Most importantly, most participants meet criteria for DMDD being one of the criterion presenting 3 or more temper outbursts per week. I wonder whether this criterion is met. It is unclear because, as metioned before, information on temper oubtursts is not transmitted fully.*

Critically, the assessment of temper outbursts and mood for a DMDD diagnosis using a semi-structured interview (i.e., K-SADS) differs dramatically from in vivo naturalistic reporting in real time. Thus, a discrepancy is expected to some extent. Indeed, while the children and parents endorsed a history of severe and impairing temper outbursts at least 3 times a week and other DMDD symptoms during the clinical assessment, when measured prospectively via EMA it may differ due to many reasons including missing prompts, specific context/timing of one week of their daily life, or the effect of completing the EMA and being reminded to reflect on one's/one's child's mood repeatedly.

*10. As suggested by the referee, 3 decimal reporting is not customary. APA guidelines suggest 1 decimal for Means and SD of integer scales, 2 decimal for other statistics, 2 or 3 decimal for exact p-value unless p<.001.*

Thank you for referring us to the APA guidelines. We find these guidelines reasonable and have modified our reporting of figures to better align to them.

*11. In the current point 2.3.2 Data collection procedure, there is confusion on the reporting of ages. The referee also mentions this section, because it clearly leads to confusion. The sentence is written in future tens, because describes future data collection. How is therefore possible to report the Mean age and SD and % of males of the future participants? These numbers belong to the preliminary data only.*

Thank you for your suggestion! We agree that these numbers belong to the preliminary data and now only report them on page 7, in the new section 2 which is dedicated to the preliminary data.

*12. Why the confirmatory dataset is expected to collect approximately 300 data points from 20 participants if these are collected using 3 prompts over 7 days? Wouldn’t be this close to 420 data points? Or this is estimation based on the % of missing data points in the preliminary sample that the authors do not report?*

Thank you for your attention to our rationale for planned sample size. You are correct, the estimated sample size is based on the rate of missing prompts. We now report the percentage of missing prompts in Table 1a on pages 9-10.

*13. The rationale for prioritizing a population-level prediction approach should be provided way in advance in the introduction. All this text “we prioritized developing a system with no requirement for individualized training can deliver useful interventions. Furthermore, we have seen in the literature that a population-level approach can indeed produce generalizable predictions from EMA data [21, 22] and that even in cases where personalized prediction is feasible, that population-level approaches can achieve similar performance [20].” Should be provided in the background, instead of nearly at the end of the manuscript.*

We agree with the reviewer’s suggestion and have now moved that discussion to the introduction, on pages 4-5.

*14. Under sample size rationale of current point 3.2 it is said that “our preliminary data had 12 participants with 1 week of data, and 39 participants with 2 weeks of data.”. This is unclear; I thought that data was collected over 7 days only, but here 2 weeks are mentioned.*

Thank you for this question and apologies for the confusion. The mention of participants with 2 weeks of data was a mistake on our part, as after double-checking, we see that only 1 week of data per participant was used for all of our preliminary data analyses. We have corrected and clarified the amount of data per participant on page 7:

"We analyzed a preliminary set of data collected starting July 2018 and ending in December 2022, in which n=57 participants (with a diagnosis of either ODD, DMDD, or sub-threshold DMDD) completed EMA, with a total of 932 prompts (3 prompts per day for 7 days, up to 21 completed prompts per participant depending on completion rate)."

*15. The current point 3.5 Correlational analysis of preliminary data, which provides Figure 1 with the colored matrix, it is unclear why this analysis, which is not declared when describing statistical approaches, is carried out. The section says that “During the model development process, we examined the pairwise correlations between variables in order to identify pairs of variables that were likely to be redundant. We found that "happy" and "unhappy" were highly negatively correlated, and that "unhappy", "grouchy" and "frustrated" were highly positively correlated, as we show in Fig 1.” But that’s it. So what is the conclusion? Are these redundant or not? If they are, what are the authors going to do about it? Authors should declare the analyses, justify it, and then interpret the results.*

Thank you for this suggestion! Initially, we included this figure because we thought it could enhance the intuition behind our reported results. However, we realize from your comment that the figure adds confusion to the paper. We have now removed the figure in order to tighten the focus of the paper on the preliminary analysis and the protocol for the confirmation study.

Sincerely,

Charles Zheng, Ph.D.

Machine Learning Team

Functional Magnetic Resonance Imaging Facility

National Institute of Mental Health, National Institutes of Health

9000 Rockville Pike, Building 10, Room 3D39

Bethesda, MD 20892-2670 USA

E-mail: [charles.zheng@nih.gov](mailto:charles.zheng@nih.gov)
